# Nasal Residence Depending on the Administered Dosage Form: Impact of Formulation Type on the In Vivo Nasal Retention Time of Drugs in Rats

**DOI:** 10.3390/pharmaceutics17070863

**Published:** 2025-06-30

**Authors:** Daisuke Inoue, Yoshihiro Seto, Hideto To

**Affiliations:** 1 Department of Medical Pharmaceutics, School of Pharmacy and Pharmaceutical Sciences, University of Toyama, 2630 Sugitani, Toyama 930-0194, Japan; yoshseto@pha.u-toyama.ac.jp (Y.S.); hidetoto@pha.u-toyama.ac.jp (H.T.); 2 Molecular Pharmaceutics Laboratory, College of Pharmaceutical Sciences, Ritsumeikan University, 1-1-1 Noji-higashi, Kusatsu Shiga 525-8577, Japan

**Keywords:** nasal absorption, intranasal administration, nasal residence time, mucociliary clearance, nasal formulation, formulation development, intranasal drug delivery

## Abstract

**Background/Objectives**: The precise control of drug absorption through the nasal mucosa following intranasal administration can be achieved through optimal formulation development that considers the nasal retention properties of the administered dosage form. This study aimed to quantitatively elucidate the effect of formulation type on nasal residence time in vivo. **Methods**: The nasal residence behavior of various formulation types, including solutions, particulates, and powders, was estimated in rats. Furthermore, the effect of mucoadhesive polymers on the nasal residence time was investigated using gel and powder dosage forms of sodium alginate. **Results**: The nasal retention behavior of the formulation in the nasal cavity differed depending on the dosage form. The polystyrene microparticles and lactose powder, a non-adhesive powder, were quickly eliminated into the nasopharynx, whereas the solution remained in the nasal cavity longer than the other formulations. The clearance behavior of the solution was investigated, and it was found that the solution was quickly transported to the stomach without being retained in the esophagus. The disappearance of the gel and powder with the mucoadhesive polymer was different, with the powder clearing faster. This difference in clearance is thought to be due to the powder being cleared before dissolving and diffusing into the nasal mucus. **Conclusions**: It has been clearly shown that the nasal residence behavior differed depending on the dosage forms. The addition of mucoadhesive polymers was effective in improving the nasal residence of the drug, and more-effective formulations for nasal application can be developed by combining optimal dosage forms, such as powders and gels.

## 1. Introduction

The nasal administration route has attracted attention for the rapid and high absorption of various drugs, not only compounds that are poorly absorbed orally [1,2] but also larger molecular modalities such as peptides [3,4,5], nucleic acids [6,7,8], proteins [9,10,11,12], and antibodies [13,14]. In addition, the potential for an efficient drug delivery strategy that utilizes a direct delivery route from the nose to the brain has led to developmental challenges in the treatment of central nervous system (CNS) diseases [15,16,17]. For several decades, numerous studies have reported the advantages of intranasal administration for systemic absorption and brain drug targeting, indicating the possibility of therapeutic effectiveness for various diseases in rodent models [18,19,20,21] and patients [22,23,24,25]. On the other hand, formulation development for intranasal application has been investigated by designing various novel formulations and dosage forms, including solutions for spraying [26,27], viscous formulations such as mucoadhesive gel [28,29,30,31,32], nano/micro-sized particulates [33,34], and powders [35,36,37]. Even today, when such active research and development is being considered, a few formulations that are expected to be absorbed systemically are used for practical purposes, and there are currently no pharmaceuticals in the market that have been clinically validated to be transported directly to the brain or CNS region. To date, many clinical trials using nose-to-brain drug delivery systems for CNS disorders have been conducted [4], and nasal formulations for several diseases, such as migraine, diabetes insipidus, endometriosis, and hypoglycemia, have been approved [38]. The most important milestone for the wide adoption in practice of nasal formulations for various diseases is to consistently demonstrate in patients the high drug concentrations in target tissues and efficient therapeutic effects indicated in animal models following intranasal application. The approach that can be taken from the perspective of formulation development to address the challenging issue of maximizing therapeutic efficacy in humans can be to accurately understand the physiological function of the nose and elucidate methods for formulation optimization that precisely control drug absorption and tissue targeting considering this physiological function.

**Figure 1:**
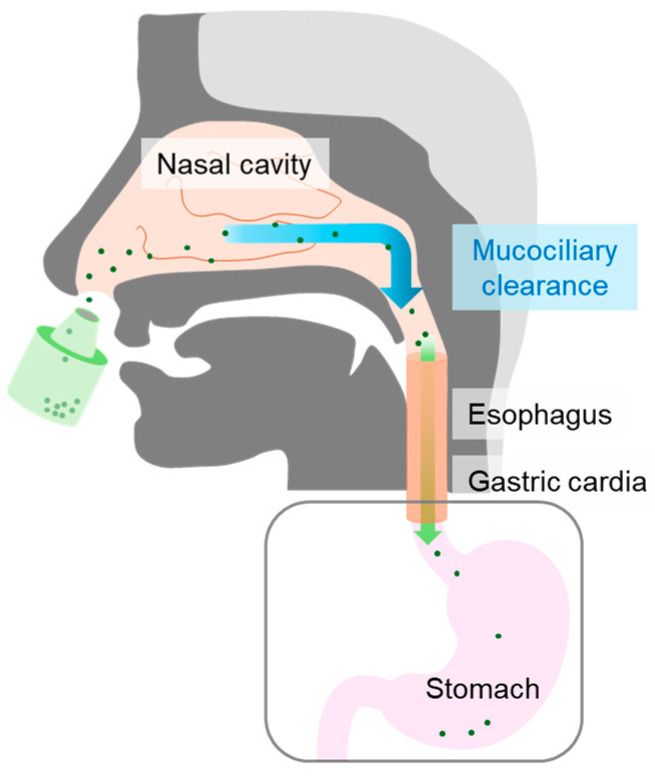
Schematic of the drug translation pathway following intranasal administration.

Nasal mucociliary clearance (MC) is a well-known physiological function of the nasal cavity that affects drug absorption following intranasal administration [39,40,41]. Due to MC, a physiological function of non-specifically eliminating foreign substances, the formulations and drugs applied to the nasal cavity are also translated to the pharynx in a relatively short time. Subsequently, the drug passes through the esophagus and reaches the stomach, where it is absorbed from the gastrointestinal tract (Figure 1).

Previous reports have demonstrated that drug solutions that do not have mucoadhesive properties are cleared from the nasal cavity within 15–30 min by MC [42,43]. As a formulation strategy to effectively improve the nasal retention time of drugs by avoiding rapid clearance by MC, the addition of mucoadhesive substances or the development of various dosage forms has been investigated, including viscous gels [44] and powders [45]. Some behavioral characteristics of formulation deposition and dispersion on the mucosa, drug dissolution and diffusion from the formulation, and residential pattern of the drug in the nasal cavity would differ depending on the dosage forms applied intranasally. Various nasal formulations have been developed previously, and the efficacy of each formulation has been evaluated using different in vitro/in vivo systems for the distribution pattern on the nasal cavity, residence time, and drug dissolution into mucus after intranasal administration. When comparing various formulations objectively and quantitatively with reference to previous reports, it is difficult to accurately determine which dosage form or formulation is more useful based on the formulation characteristics.

In the present study, the nasal residence behavior for different dosage forms (e.g., solutions, powders, and particulates) was investigated by using an in vivo evaluation method to compare individual characteristics. Furthermore, the effect of mucoadhesive polymers on the nasal residence pattern in vivo in different dosage forms, including powders and gels, has been elucidated.

## 2. Materials and Methods

### 2.1. Materials

#### 2.1.1. Regents

Fluorescent microspheres (FMS; Fluoresbrite YG microspheres, particle diameter 6.00 μm) were purchased from Polysciences, Inc. (Warrington, PA, USA). Phosphate-buffered saline (PBS; pH: 7.4) was purchased from Nacalai Tesque (Kyoto, Japan). Fluorescein isothiocyanate–dextran (average molecular weight of 70,000, FD-70) and sodium alginate (from brown algae, low viscosity) were purchased from Sigma-Aldrich (St. Louis, MO, USA). Lactose monohydrate, carboxymethyl cellulose sodium salt (CMC-Na), and (±)-Dithiothreitol (DTT) were purchased from FUJIFILM Wako Chemical Corporation (Osaka, Japan).

#### 2.1.2. Animals

Male Wistar rats weighing 250–300 g were used in all the animal experiments. All animal studies were conducted under guidelines approved by the local Animal Care Committee of Ritsumeikan University in accordance with the Principles of Laboratory Animal Care (NIH publication #85-23). The animals were kept under conditions of food and water intake ad libitum, at a room temperature of 24 ± 1 °C, humidity of 55 ± 5%, and a light/dark cycle of 7:00 to 19:00. Experiments were performed using three to five independent animals for each experimental condition.

### 2.2. In Vivo Study for Evaluating the Effect of Drug Application Site on Nasal Residence Time

#### 2.2.1. In Vivo Nasal Residence Study

The intranasal retention of formulations over time was estimated by determining the remaining amount of non-absorbable markers, using fluorescent dextran (70 kDa) for solutions and powders, and a fluorescent microsphere was used as the particulate dosage form. Nanosized particles were considered inappropriate because nanoparticles can penetrate the mucosal membrane or be taken up into the brain in the particle form. Therefore, in this study, we used microsized particles, which are also commonly developed for nasal formulations, as a non-absorbable marker for the particle dosage form. The composition of each dosage form is listed in Table 1. The concentration of FD-70 in each formulation was optimized to measure, fluorometrically, excitation/emission = 444 nm/500 nm (RF-5300PC, Shimadzu, Kyoto, Japan). The suspension of fluorescent microspheres was diluted 10,000 times with PBS for dosing. The nasal residence times of the formulations were evaluated as previously described [43]. Under isoflurane anesthesia (introduced at 4%, maintained at 2%, inhalation), rats were administered each formulation (5.0 µL for solutions, particulates, and gels; 0.5 mg for powders) and maintained an anesthetic condition until the sampling procedure. The residual marker in the nasal cavity was collected at intervals of 5, 10, 15, 20, 30, 60, 120, and 240 min. Cannulation was performed according to the method of Hirai et al. [46], in which the rat skin was incised to expose the esophagus and a cannula was inserted from the esophagus to the nasopharynx. To completely wash out the markers, PBS and 50 mM DTT solution (each 5 mL) were passed through the nasal cavity from the cannula once and twice, respectively. The amount of markers in the collected samples was determined from fluorescence intensity (excitation/emission = 444/500 nm and 441/486 nm for FD-70 and FMS, respectively).

#### 2.2.2. Impact of Drug Deposition Sites in the Nasal Cavity on Nasal Residence

To evaluate the effect of the sites where drugs are deposited in the nasal cavity on the nasal residence, the nasal retention time of drugs was evaluated after an aliquot of FD-70 solution (5 µL) was applied intranasally to rats held in a fixed position. Under isoflurane anesthesia, the rats were fixed in the prone, right-facing, left-facing, or supine position to apply the drug solution to the upper side, nasal septum, lateral wall, and bottom side, respectively, and 5 μL of drug solution was instilled 10 mm from the left nostril. The nasal retention profiles of the drug were determined in the same manner as described above. Fourteen rats were used for the upper, septum, and lateral wall (n = 4 for 15 and 30 min, n = 3 for 60 and 120 min), and 20 rats were used for the bottom (n = 5 at each sampling point).

#### 2.2.3. Transfer Dynamics of Drugs After Disappearance from the Nasal Cavity

Following intranasal application, the drugs or formulations are translated to the pharyngeal side by MC, after which the drugs undergo gastrointestinal absorption. The kinetic profile of the drug cleared from the nasal cavity into the stomach was evaluated. Under isoflurane anesthesia, rats were kept in the prone position, and 5 µL of FD-70 solution was applied intranasally. After appropriate intervals (5, 15, 30, and 60 min), the esophagus was harvested, washed with PBS and 50 mM DTT (each 5 mL), and the residual amount of FD-70 in the esophagus was determined fluorometrically. To estimate the amount of drug translated into the stomach, after the gastric cardia was ligated, 5 µL of FD-70 solution was applied intranasally, and the FD-70 remaining in the esophagus was recovered using PBS and 50 mM DTT at time intervals of 15, 30, 60, and 120 min. The esophagus and stomach were harvested from independent rats. Twelve rats were used for the esophagus and stomach (n = 3 at each sampling point).

### 2.3. Effect of Formulation Type on the Nasal Residence of Drugs

#### 2.3.1. Impact of Dosage Forms on Nasal Retention Time

The in vivo nasal residences of the three dosage forms (solutions, powders, and particulates) were evaluated to clarify the differences in nasal retention time depending on formulation type. The dosing formulations were prepared as 5 μL of PBS containing 2.0 *w*/*v*% FD-70 for the solution, 0.5 mg of a mixture of 10% FD-70 in lactose for the powder (Powder/Lac), and 5 μL of a 10,000-fold diluted FMS with PBS for the particulate, and each formulation was administered intranasally to rats. Twenty-two rats were used for the Powder/Lac (n = 4 for 5, 15, 20, and 30 min, n = 3 for 60 and 120 min), and 26 rats were used for the particulate (n = 5 for 5, 10, 15, and 30 min, n = 3 for 60 and 120 min).

#### 2.3.2. Effect of Mucoadhesive Polymers on Nasal Residence in Powder Formulation

Mucoadhesive polymers have been widely used to improve the nasal residence properties of drugs [47]. The effects of mucoadhesive polymers (CMC-Na and sodium alginate) on the nasal retention time of the formulations were investigated. Powders were prepared by mixing 10% FD-70 with CMC-Na or sodium alginate (Powder/CMC and Powder/Alginate), and Powder/Lac was used as a non-adhesive powder control. Seventeen rats were used for Powder/CMC (n = 4 for 15 and 30 min; n = 3 for 60, 120, and 240 min), and 19 rats were used for Powder/Alginate (n = 5 for 15 and 30 min; n = 3 for 60, 120, and 240 min).

#### 2.3.3. Impact of Dosage Forms on Nasal Residence in Mucoadhesive Formulation

The dosage form of the formulations may affect their mucoadhesive properties. Even if the same mucoadhesive polymer is added, the nasal residence of drugs may differ depending on the dosage form. The gel form of sodium alginate was used for comparison with the powdered form. FD-70 was dissolved in PBS containing 2% sodium alginate to form a gel. Sixteen rats were used for Gel/Alginate (n = 4 for each time point).

### 2.4. Analytical Procedure

#### 2.4.1. Calculation of Drug Disappearance Rate Constant by Mucociliary Clearance

We previously established an evaluation methodology for determining the rate constant of drug disappearance from the rat nasal cavity by MC [43]. The time profile of the drug remaining in the nasal cavity showed biphasic elimination and the initial clearance (α-phase) was attributed to MC. Consequently, the disappearance rate constant by MC (*k*_mc_) was determined by the disappearance rate constant calculated from the slope of first-order elimination (up to 30 min after drug administration).

#### 2.4.2. Statistical Analysis

Data are expressed as the mean ± S.E.M. (n = 3–5 for each experimental condition). Statistical significance was determined based on multiple comparisons with Dunnett’s test using SPSS software (IBM SPSS Statistics, version 29.0.0.0, IBM Corporation, Armonk, NY, USA).

## 3. Results

### 3.1. In Vivo Nasal Residence of Drugs Following Intranasal Application

#### 3.1.1. Differences in Nasal Residence Depending on the Site of Drug Deposition

**Figure 2:**
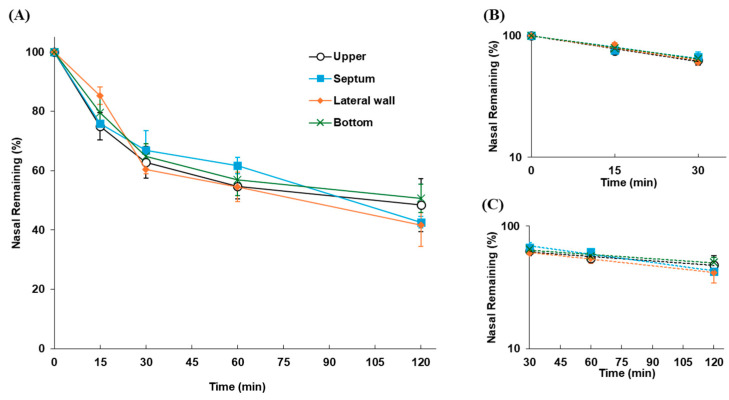
Disappearance of FD-70 following its application at different positions in the nasal cavity. (**A**) Time profiles of FD-70 remaining in the nasal cavity. (**B**) Slope of the linear elimination of the α-phase (up to 30 min) plotted on a semi-logarithmic scale. (**C**) Slope of the linear elimination of the later phase (30–120 min) plotted on a semi-logarithmic scale. Data are expressed as the means of three–five independent experiments, with vertical bars showing S.E.M.

The nasal residual profiles of FD-70 following application as the solution to rats at different locations in the nasal cavity are shown in Figure 2. The clearance profile of FD-70 was consistent regardless of whether it was applied to the upper, nasal septum, lateral wall, or bottom positions in the nasal cavity. The *k*_mc_ values of FD-70 were 0.0162, 0.0144, 0.0155, and 0.0146 min^−1^ for the upper, septum, lateral wall, and bottom, respectively, and no significant differences between these values were observed (Table 2).

#### 3.1.2. Kinetics of FD-70 After Disappearance from the Nasal Cavity

**Figure 3:**
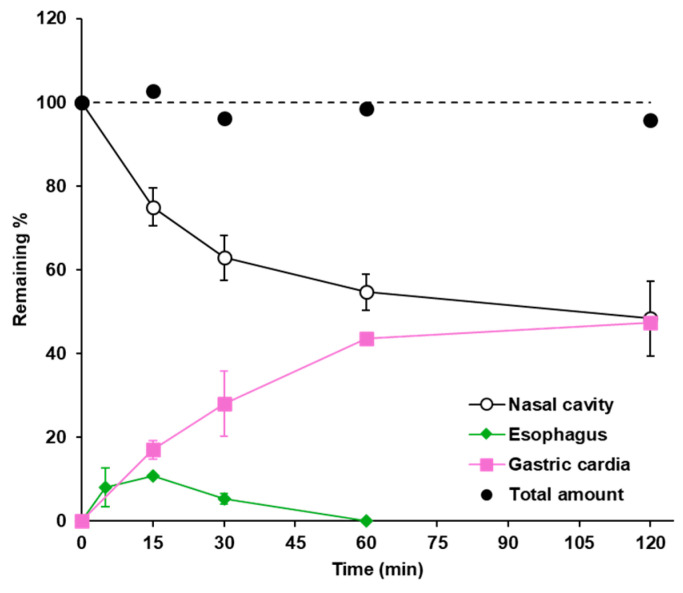
Drug transition from the nasal cavity to the stomach. Time profiles of FD-70 in the nasal cavity, esophagus, and gastric cardia. The cumulative amount of FD-70 in the three parts is shown as a solid black circle. Data are expressed as the means of three–five independent experiments, with vertical bars showing S.E.M. The dotted line represents the remaining amount of 100%.

The time profiles of the residual FD-70 amount in the nasal cavity, esophagus, and gastric cardia after intranasal administration are shown in Figure 3. The cumulative amount of FD-70 in the three parts is plotted as solid circles. It was found that FD-70, which disappeared from the nasal cavity, quickly translated to the esophagus and passed to the stomach without being retained in the esophagus. The cumulative amount of FD-70 remained at almost 100%, indicating that the drug cleared from the nasal cavity quickly, passed through the esophagus, and was translated into the stomach.

### 3.2. Impact of Formulation Types on the Nasal Residence of Drugs

#### 3.2.1. Impact of Dosage Forms on the Nasal Residence of Formulations

**Figure 4:**
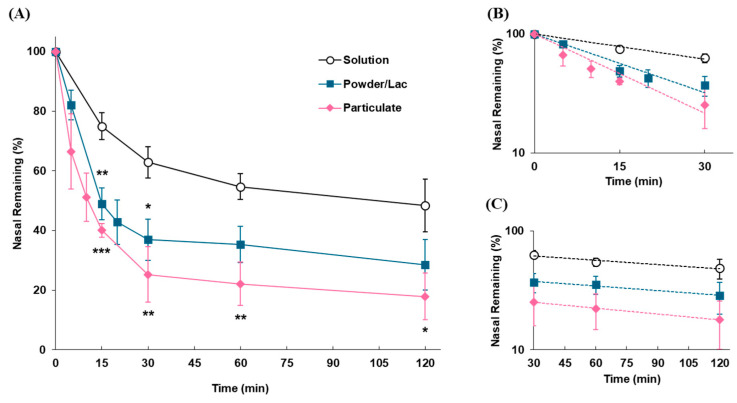
Disappearance profiles of each dosage form in the nasal cavity following intranasal administration. (**A**) Time profiles of remaining formulations in the nasal cavity. (**B**) Slope of the linear elimination of the α-phase (up to 30 min) plotted on a semi-logarithmic scale. (**C**) Slope of the linear elimination of the later phase (30–120 min) plotted on a semi-logarithmic scale. Data are expressed as the means of three–five independent experiments, with vertical bars showing S.E.M. Statistical significance is represented as ***, *p* < 0.001; **, *p* < 0.01; and *, *p* < 0.05, compared with the solution.

The clearance profiles of each dosage form in the nasal cavity following intranasal administration in rats are shown in Figure 4, demonstrating that the particulate and powder forms were eliminated from the nasal cavity significantly more rapidly than the solution. The disappearance rates from the nasal cavity caused by MC (*k*_mc_) for the particulates and powder were higher than those for the solution. The half-lives of elimination for these dosage forms were 13.6 and 18.3 vs. 42.8 min by MC, and 183.4 and 233.4 vs. 249.3 min in the later phase, respectively (Table 3).

#### 3.2.2. Effect of Mucoadhesive Polymers on Nasal Residence of Powder Formulations

**Figure 5:**
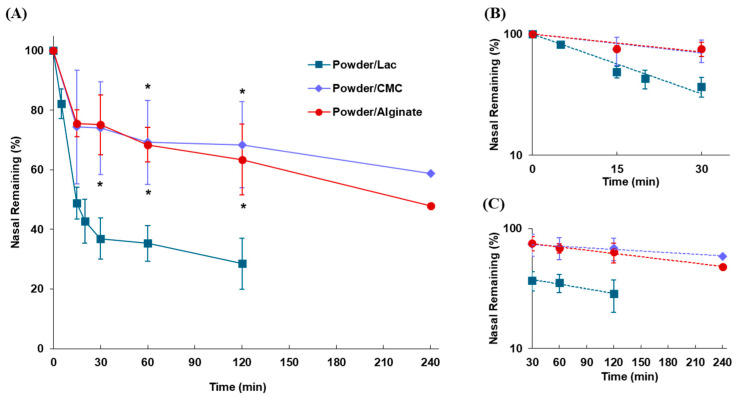
Disappearance profiles of mucoadhesive powers in the nasal cavity following intranasal administration. (**A**) Time profile of the powder remaining in the nasal cavity. (**B**) Slope of the linear elimination of the α-phase (up to 30 min) plotted on a semi-logarithmic scale. (**C**) Slope of the linear elimination of the later phase (30–120 min) plotted on a semi-logarithmic scale. Data are expressed as the means of three–five independent experiments, with vertical bars showing S.E.M. Statistical significance is represented as *, *p* < 0.05, compared with the Powder/Lac.

The effects of adding CMC-Na and sodium alginate on the disappearance of powder formulations after intranasal administration were evaluated. The clearance profiles of the powders after intranasal administration to rats are shown in Figure 5. The nasal retention was significantly improved by the addition of CMC-Na and sodium alginate to the powder formulations, revealing that approximately half of the administered dose (58.7% and 48.0% for CMC-Na and sodium alginate, respectively) remained in the nasal cavity for up to 240 min. The magnitude of the polymer effect, such as the mucoadhesive and viscosity-increasing effects, was similar for both polymers, and the nasal retention time contributed by MC (*k*_mc_ and *t*_1/2(mc)_) increased by 3.15 and 3.32 times for Powder/CMC and Powder/Alginate, respectively (Table 4).

#### 3.2.3. Impact of Dosage Forms on Nasal Residence for Mucoadhesive Formulations

**Figure 6:**
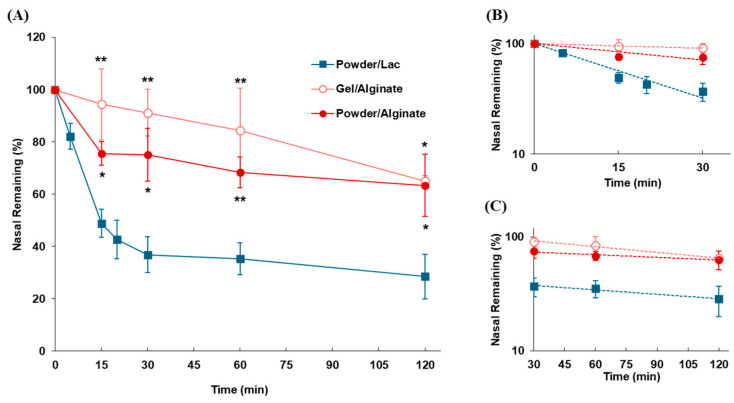
Disappearance profiles of mucoadhesive gel and power in the nasal cavity after intranasal administration. (**A**) Time profile of formulations remaining in the nasal cavity. (**B**) Slope of the linear elimination of the α-phase (up to 30 min) plotted on a semi-logarithmic scale. (**C**) Slope of the linear elimination of the later phase (30–120 min) plotted on a semi-logarithmic scale. Data are expressed as the means of three–five independent experiments, with vertical bars showing S.E.M. Statistical significance is represented as ** *p* < 0.01 and * *p* < 0.05, compared with Powder/Lac.

Gel and powder formulations containing sodium alginate (Gel/Alginate and Powder/Alginate) were prepared, and their nasal residences in rats were compared (Figure 6). The retention time of the gel formulation in the nasal cavity was higher than that of the powder formulations. An increase in the residual amount of the formulations in the nasal cavity was observed by applying sodium alginate gel, although the difference was not statistically significant compared to that of the sodium alginate powder. The nasal residence of formulations was improved by the gelation of sodium alginate; the retention time of formulations undergoing MC (*t*_1/2(mc)_) was increased 11.8 and 3.56 times compared to the non-adhesive powder (Powder/Lac) and mucoadhesive powder formulation (Powder/Alginate), respectively (Table 5).

## 4. Discussion

Nasal formulations have many attractive advantages, such as high drug absorption, rapid onset of action, avoidance of gastric digestion and hepatic first-pass elimination, and efficient drug delivery to the brain and CNS regions, suggesting that formulation development for intranasal applications will progress in the future. However, many challenges remain for practical applications, such as precise control of the dissolution behavior of the formulation in the nasal cavity and drug absorption through the nasal mucosa, the development of optimization systems for formulation dosage forms suitable for various drug modalities, and the avoidance of toxicity and irritation, including an unacceptable formulation pH and the inclusion of irritating substances. Although there are many reports that individually evaluate the effects of each additive on the development of novel nasal formulations, a comprehensive evaluation of the extent to which changes in the formulation dosage forms contribute to nasal residence using a single evaluation system has not been reported. Herein, we focused on nasal residence, which is important for the development of nasal formulations and contributes greatly to drug absorption after intranasal administration. Kinetic evaluation of the formulations disappeared by MC and detailed quantitative observations of the nasal retention behavior were conducted to clarify how the formulation dosage forms are related to MC.

In this study, we used a previously established system to evaluate the in vivo nasal residence time of drugs [39]. In this method, the test was conducted under anesthesia to quantitatively evaluate the nasal retention behavior of the drug by MC. In a previous study, we evaluated the effect of anesthetics used in this experimental system on the drug disappearance rate of MC, and it was revealed that the disappearance rate under anesthetized conditions was reduced to approximately 80% compared to non-anesthetized conditions. In the evaluation of nasal residence time under non-anesthesia and free-moving conditions in rats, in addition to the simple disappearance behavior by MC, the influence of some factors, including air movement due to respiratory fluctuations and changes in posture due to movement, are greatly affected by the nasal residence of the drug. As a result, it is difficult to evaluate the pure disappearance behavior of MC under non-anesthetic conditions. Therefore, in the present study, it was necessary to capture detailed changes in MC behavior, such as differences in the deposition site and comparisons between formulations, as it is thought that this cannot be examined under non-anesthesia. The study was conducted under isoflurane anesthesia, which can maintain a constant state of anesthesia.

The solution and gel formulations were applied 10 mm away from the nostrils. In rats, 10 mm from the nostril is the center of the nasal cavity, and the marker can be instilled into the airway mucosa where cilia are densely expressed, allowing for the accurate evaluation of the effects of MC. However, if the marker is dropped just a short distance from the nostril, it is dropped into the nasal vestibule, where there are no ciliated cells, and the applied marker will remain for a very long time. The residual amount of marker remains because it does not receive ciliary movement, and if the residual amount is included it will be impossible to accurately evaluate the effects of MC. Therefore, in the present study, the position where the solution and gel were applied was strictly set at the center of the nasal cavity (10 mm from the nostril).

The site of intranasal deposition of the administered formulation greatly contributes to absorption and brain delivery efficiency [48,49]. In the development of nasal formulations, the intranasal distribution of the administered formulations is evaluated during formulation optimization, and the development of novel dosing devices that allow the formulation to be deposited at a target site in the nasal cavity (such as the area around the olfactory region or the entire airway mucosa) is considered in parallel [50,51]. Different intranasal deposition sites of the formulation can cause variable drug disappearance behavior by MC, resulting in fluctuations in drug absorption or brain uptake. In this study, a previously established estimation system for in vivo nasal residence in rats [39,43] was used to quantitatively evaluate the in vivo nasal retention times for several dosage forms. To accurately characterize nasal residence time, detailed characteristics of the formulation disappearance profile by MC in the system were validated. First, the site dependency of the disappearance of intranasally applied formulations by MC was evaluated. It is known that the disappearance of foreign substances from the nasal cavity has a biphasic profile. It is generally assumed that the initial rapid phase (α-phase) is based on MC, and that the later slow phase (β-phase) is attributable to other factors [52]. Similarly, our evaluation system revealed that the disappearance of drug markers from the nasal cavity exhibited a biphasic pattern [43]. Therefore, in our system, the disappearance rate constant of the formulations by MC (*k*_mc_) was calculated from the initial elimination phase (α-phase), and the fluctuation of *k*_mc_ (and elimination half-life, *t*_1/2 (mc)_) was quantitatively evaluated. The disappearance of the formulation after the application of the FD-70 solution to the rat nasal cavity was consistent, regardless of whether the solution was applied to the upper, nasal septum, lateral wall, or bottom parts (Figure 2). No differences in disappearance rates were observed in the application site dependency when the calculated *k*_mc_ values were compared (Table 2). MC occurs when cilia present throughout the nasal mucosa are bundled together and repeatedly move back and forth at a constant rhythm, gradually shifting the thin mucus blanket on the surface of the mucosa toward the pharynx by ciliary movement [53]. This MC mechanism suggests that the formulations applied intranasally disappear by MC, which functions uniformly throughout the nasal mucosa; therefore, the disappearance rate caused by MC cannot fluctuate even when the site of application in the nasal cavity is varied. It was clarified that formulations are functionally excluded by the MC mechanism, regardless of the site of deposition in the nasal cavity. In the present study, when the solution was applied to the center of the nasal cavity, it was widely dispersed over the surface of the airway mucosa, and the MC functioned throughout the nasal cavity, which is thought to have functioned as a complete clearance mechanism. For example, for direct drug delivery to the brain, it is necessary to deposit it in the olfactory region; therefore, the intranasal deposition site, which is in contact with the olfactory region, is an important factor for achieving effective brain drug delivery. In addition, in cases where the application site cannot be strictly set as in this study, if the dropped or sprayed formulation is deposited on the nostrils, paranasal sinuses, or near the nasopharynx, it may result in insufficient clearance by MC or insufficient mucosal contact of the drug, which may affect drug absorption. Although it is premature to conclude that the site of deposition has no effect on nasal residence, our findings suggest that the physiological function of MC throughout the nasal cavity works in an integrated manner with the mucus layer present over a relatively wide range of mucosal surfaces.

The formulations cleared from the nasal cavity to the pharynx pass through the esophagus to the stomach, and it is assumed that a portion of the formulation is absorbed through the gastrointestinal mucosa, similar to oral administration. However, there are no reports on the kinetic behavior of drug transport from the nasal cavity to the gastrointestinal tract. Therefore, we performed kinetic analysis of the translation of the formulations into the stomach after intranasal administration. Our results demonstrated that the intranasal drug gradually disappeared from the nasal cavity, and the cleared drugs rapidly passed through the esophagus and reached the stomach (Figure 3). The amount of drug remaining in the esophagus was small at all sampling times and was eliminated within 60 min after administration, suggesting that the drug cleared from the nasal cavity did not remain in the esophagus and was rapidly transferred to the stomach. In addition, the total amount of drug remaining in the nasal cavity, esophagus, and stomach was always almost equal to the administered amount, indicating that the drug administered into the nasal cavity was cleared by MC and that all of it reached the stomach via the esophagus. Because it was thought that digestion in the stomach would have a large effect on the quantitative evaluation of drug residence, in this study, instead of directly measuring the intragastric concentration of the marker, an indirect evaluation was adopted in the gastric cardia, which is the part just before the drug enters the stomach; ligation was carried out, and the amount of drug remaining in the cardia was quantified. The obtained results do not require consideration of digestion in the stomach, and the total amount estimated to enter the stomach can be measured. Based on these results, it may be possible to accurately predict the total amount of drug absorbed after intranasal administration by evaluating the amount absorbed through the nasal mucosa and by considering the rate of drug absorption after oral administration. These findings will be beneficial for constructing a prediction model for drug absorption after intranasal administration from in vitro systems.

In nasal formulation development, a variety of dosage forms have been considered, including droplets/liquids, such as sprays, mists, and viscous gels with polymers [28,29,30,31,32,44]; powders, such as mucoadhesive formulations and freeze-dried formulations [54]; and particulates in dispersed nano-/micro-ordered particles [33,34]. It is unlikely that the various formulations developed will have the same nasal residence characteristics. Even if the formulation components are similar in some dosage forms, these nasal residences can fluctuate depending on the dosage, whereas there are no reports that have compared the nasal residences of different dosage forms using the same evaluation system. Clarifying the differences between the dosage forms could provide useful information for the development of nasal formulations. The nasal residence characteristics of the solution, powder, and micro-sized particulates were evaluated to assess the differences between dosage forms. Compared with the solution, the powder and particulate were cleared more rapidly from the nasal cavity (Figure 4). The solution may diffuse widely throughout the nasal mucosa immediately after administration and is gradually cleared by MC. In contrast, the powder was expected to remain partially solid at the deposition site until it dissolved in a small amount of mucus, suggesting that it could be affected by disappearance via MC before the powder dissolved and spread sufficiently. In addition, the micronized particles disappeared most rapidly because they were more affected by MC without diffusing widely in the nasal cavity and because of the reduced adhesion due to their spherical shape. In fact, the disappearance rates from the nasal cavity caused by MC (*k*_mc_) for the particulates and powder were lower than those for the solution (Table 3). However, it should be noted that the findings were obtained with a single formulation of lactose powder or polystyrene microparticles, and the nasal retention of the formulations was largely dependent on their composition contained in that formulation. For example, it is thought that the addition of mucoadhesive polymers with powders or surface modification and nanosized microparticles can greatly improve nasal residence. It is clear that the nasal residence of formulations is affected by the dosage form, and even a simple modification in dosage form can cause significant fluctuations in nasal residence, resulting in variations in drug absorption. Therefore, this possibility should be considered in the development of nasal formulations.

Since a simple powdered formulation may be insufficient to improve the nasal residence of the drug, the strategy of adding polymeric substances with mucoadhesive properties has been frequently adopted, including cellulose derivatives [55], polyvinylpyrrolidone [56], sodium alginate [57], and chitosan [44,58]. The effect of mucoadhesive polymers on the nasal retention time of powders was evaluated using CMC-Na and sodium alginate. Mucoadhesive powder significantly increased the retention time in the nasal cavity compared with lactose powder, which has non-mucoadhesive properties (Figure 5). This result indicates that the addition of mucoadhesive substances contributes to improved nasal residence, which can be an effective strategy for improving drug absorption after intranasal administration. This suggests that the polymers increase the intranasal residence time of the drug because of the additive effect of mucoadhesiveness and the viscosity-increasing effect of each polymer. The results obtained here simply indicate that one of the properties of a polymer resulted in an increased nasal residence time of the drug, and various factors may be considered to contribute to the detailed mechanism of the polymer’s effect. To precisely evaluate the effect of a mucoadhesive polymer on the intranasal residence time of the drug, it is necessary to measure the degree of mucoadhesion and the degree of an increase in viscosity separately and analyze their correlations. However, separating the analysis for these factors within this study would make the results and discussion more complicated; therefore, we first of all decided to perform a simple comparison between the dosage forms to obtain basic findings. There are many factors that need to be considered in the polymer effect, such as the degree of mucoadhesion, the viscosity-increasing effect, electrostatic interactions with mucus, and the biodegradability of the polymer; therefore, we plan to proceed with a future project to clarify the properties and influencing factors of the polymers. Furthermore, since formulation developments utilizing the gelling ability, such as sodium alginate, have been investigated frequently, we prepared a 2% gel form and the impact of gelation on the nasal retention time of drugs was estimated via comparison with that of the powder form. Gelation tended to improve drug retention in the nasal cavity compared to the powder form, enhancing the nasal residence time by 3.31 times (Figure 6 and Table 4). Mucoadhesive powders only exhibit effective mucoadhesion once they are deposited on the nasal mucosa and dissolve in mucus. While in the gel form the polymer is gelated before dosing; it can interact with the nasal mucosa immediately after deposition in the nasal cavity, with the expectation of a longer residual effect in the nasal cavity. As the gel properties of polymeric substances differ for each substance, further studies using various gelling substances are necessary to reach a conclusion regarding the usefulness of gel formulations. Our findings demonstrated that gelation is an effective strategy, at least for sodium alginate.

## 5. Conclusions

We quantitatively clarified the effect of formulation dosage forms on nasal residence by using an in vivo evaluation system. The nasal retention time after intranasal administration differed depending on the dosage form (solutions, powders, and particulates). The addition of mucoadhesive polymers was effective in improving the nasal residence of the drug, and more-effective formulations for nasal application can be developed by combining optimal dosage forms, such as powders and gels. The findings obtained provide useful information for constructing a formulation strategy that can precisely control drug absorption after intranasal administration.

## Figures and Tables

**Table 1:** Composition of formulation dosage forms.

Dosage Form	Solvent/Additive	Marker	Marker Content	Dose per Head
Solution	PBS ^a^	FD-70 ^c^	2.0 (*w*/*v*)%	5.0 µL
Particulate	PBS ^a^	FMS ^d^	Diluted 10,000 times	5.0 µL
Powder	Lactose	FD-70 ^c^	10 (*w*/*w*)%	0.5 mg
	CMC-Na ^b^	FD-70 ^c^	10 (*w*/*w*)%	0.5 mg
	Sodium alginate	FD-70 ^c^	10 (*w*/*w*)%	0.5 mg
Gel	Sodium alginate	FD-70 ^c^	2.0 (*w*/*v*)%	5.0 µL

^a^ PBS: phosphate-buffered saline (pH: 7.4), ^b^ CMC-Na: carboxymethyl cellulose sodium salt, ^c^ FD-70: fluorescent dextran (70 kDa), and ^d^ FMS: fluorescent microspheres (*ϕ*6.00 µm, 2.5% as latex solid).

**Table 2:** Pharmacokinetic parameters on disappearance of FD-70 after intranasal administration.

Site Applied	*k*_mc_ (min^−1^)	*t*_1/2(mc)_ (min)	*k*_later_ (min^−1^)	*t*_1/2(later)_ (min)
Upper	0.0162	42.8	0.00278	249.3
Septum	0.0144	48.1	0.00518	133.8
Lateral wall	0.0155	44.7	0.00418	165.8
Bottom	0.0146	47.5	0.00264	262.6

**Table 3:** Pharmacokinetic parameters for the disappearance of formulations after intranasal administration.

Dosage Form	*k*_mc_	*t*_1/2(mc)_	*k*_later_	*t*_1/2(later)_
	(min^−1^)	(min)	(min^−1^)	(min)
Solution	0.0162	42.8	0.00278	249.3
Particulate	0.0510	13.6	0.00297	233.4
Powder/Lac	0.0378	18.3	0.00378	183.4

**Table 4:** Pharmacokinetic parameters for the disappearance of mucoadhesive powders after intranasal administration.

Powders	*k*_mc_	*t*_1/2(mc)_	*k*_later_	*t*_1/2(later)_
	(min^−1^)	(min)	(min^−1^)	(min)
Powder/Lac	0.0378	18.3	0.00297	233.4
Powder/CMC	0.0120	57.8	0.00102	679.6
Powder/Alginate	0.0114	60.8	0.00207	334.9

**Table 5:** Pharmacokinetic parameters for the disappearance of mucoadhesive formulations after intranasal administration.

Dosage Form	*k*_mc_	*t*_1/2(mc)_	*k*_later_	*t*_1/2(later)_
	(min^−1^)	(min)	(min^−1^)	(min)
Gel/Alginate	0.0032	215.3	0.00386	179.6
Powder/Alginate	0.0114	60.8	0.00207	334.9

## Data Availability

The original contributions presented in this study are included in the article. Further inquiries can be directed toward the corresponding author.

## Abbreviations

The following abbreviations are used in this manuscript:

MC	Mucociliary clearance
FMS	Fluorescent microsphere
CMC-Na	Carboxymethylcellulose
FD-70	Fluorescein isothiocyanate dextran, 70 kDa
*k*_mc_	Drug disappearance rate constant by MC (α-phase)
*k*_later_	Drug disappearance rate constant in later phase (β-phase)
*t*_1/2(mc)_	Half-life of disappearance by MC (α-phase)
*t*_1/2(later)_	Half-life of disappearance in later phase (β-phase)
